# Thoracolaparoscopic-Assisted Esophagectomy for Corrosive-Induced Esophageal Stricture

**DOI:** 10.7759/cureus.7909

**Published:** 2020-05-01

**Authors:** Raghav Nayar, Vaibhav Varshney, Sunita Suman, Subhash Soni, Naveen Kumar

**Affiliations:** 1 Surgical Gastroenterology, All India Institute of Medical Sciences, Jodhpur, IND; 2 Medical Gastroenterology, Mathura Das Mathur Hospital, Jodhpur, IND

**Keywords:** esophageal stricture, corrosive, minimally invasive esophagectomy (mie), video-assisted thoracoscopic surgery (vats)

## Abstract

Corrosive-induced stricture of the digestive tract is a dreaded complication following corrosive ingestion. When surgical reconstruction is needed, esophagectomy helps to avoid the long-term complications related to leaving behind the scarred native esophagus. We tried to ascertain the feasibility and safety of a thoracolaparoscopic-assisted esophagectomy in such a setting.

A 32-year-old male presented with corrosive-induced esophageal stricture that lead to progressive dysphagia not amenable for endoscopic dilatation. Thoracoscopic approach was used for mobilization of the scarred esophagus under vision. Laparoscopic approach was used in mobilizing the stomach and creating a conduit. Esophagogastric anastomosis was performed in the neck. The patient had an uneventful recovery postoperatively and was discharged after six days on a semisolid diet.

Thoracolaparoscopic-assisted esophagectomy can be safely performed for corrosive strictures of the esophagus. Besides improving the ease of performing the procedure, it also helps mitigate the morbidity associated with conventional open surgery in such cases.

## Introduction

Corrosive esophageal stricture necessitates surgery when endoscopic measures fail or are not feasible. The diseased esophagus is usually bypassed or resected with replacement by stomach, colon or jejunum. Dissection for esophagectomy is fraught with difficult dissection of a scarred esophagus and risk of bleeding and injury to adjacent vital mediastinal structures. Resection of the esophagus is a less favorable option and a bypass is usually preferred, in view of the morbidity associated with esophagectomy [1]. However, leaving behind the native esophagus increases the risk of mucocele formation and malignant transformation. Hence, performing an esophagectomy with thoracoscopic approach can overcome the possible procedural complications and avoid long-term problems associated with retained esophagus [2]. Here, we present a case of corrosive-induced esophageal stricture in a young male, who was managed by performing thoracoscopic esophagectomy with laparoscopic-assisted gastric pull-up, with an emphasis on operative technique and its advantages.

## Case presentation

**Figure 1:**
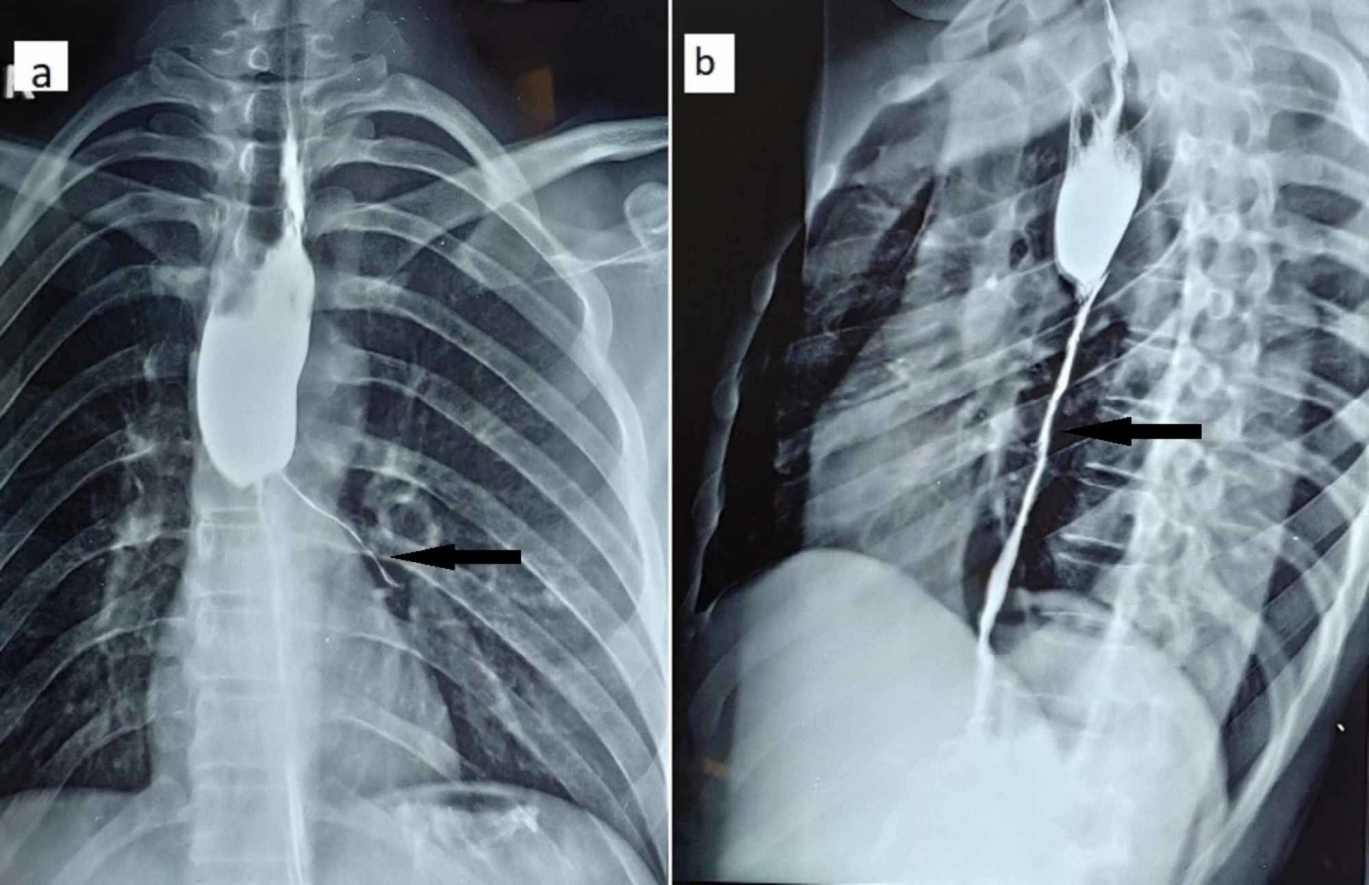
Barium esophagogram Depicting a long-segment esophageal stricture in mid and lower esophagus with contrast hold up proximal to it. (a) Anteroposterior and (b) lateral views.

A 32-year-old male presented to our department with an alleged history of accidental acid ingestion seven months back. Initially, his Esophagogastroduodenoscopy (EGD) was suggestive of Zargar grade III esophageal injury and he was managed conservatively. Over the subsequent six months, he developed progressive dysphagia and was able to swallow liquids only (grade IV). His barium swallow and EGD were suggestive of long-segment esophageal stricture (~10 cm) starting around 24 cm distal to incisors (Figure 1). Endoscopic dilatation was attempted but with subsequent failure, he was planned for surgery.

**Figure 2:**
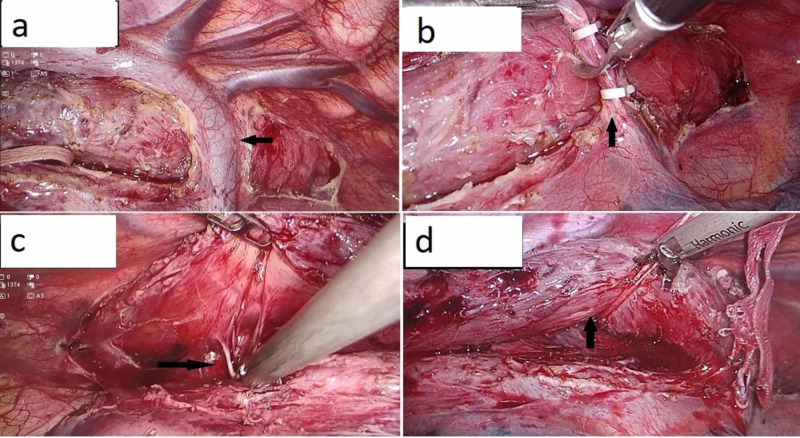
Thoracoscopic phase Displaying (a) dissected esophagus with the azygous vein, (b) dividing the clipped azygous vein, (c) dense periesophageal adhesions encountered during dissection, (d) dissected esophagus slung on an umbilical tape.

Thoracoscopic esophagectomy was carried out in the prone position with the use of a double-lumen endotracheal tube (DLT) for intubation. Three ports were placed on the right side of chest: a 12-mm port in the seventh intercostal space (ICS) in the mid-axillary line; two 5-mm ports in the ninth and fifth ICS along the posterior axillary line, respectively. After deflating the right lung using a DLT, we encountered periesophageal adhesions in the mid and lower esophagus with a dilated upper thoracic esophagus. Thoracoscopic mobilization of the esophagus was done under vision using blunt and sharp dissection. The plane of resection was kept close to the esophagus taking care to avoid injury to both the trachea and bronchus; however, the azygous vein was divided for easy mobilization (Figure 2). After the complete mobilization of the esophagus, a 28 Fr intercostal drainage tube was placed through the 12-mm camera port and rest of the port sites were closed.

**Figure 3:**
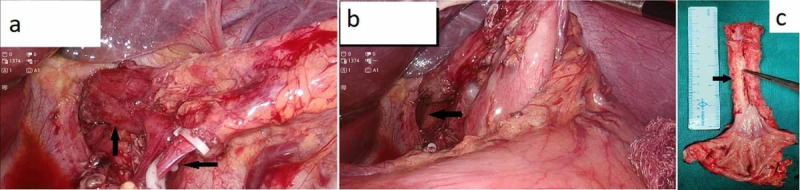
Laparoscopic abdominal phase Showing (a) dissected and clipped left gastric artery, (b) mobilized stomach and lower end of esophagus, (c) cut open specimen showing the long-segment stricture.

For the abdominal phase, the patient was placed supine with legs split and in a slight reverse Trendelenburg position. A 12-mm port was placed infraumbilical, two 5-mm ports were placed in the right and left mid-clavicular line at the level of umbilicus, and another assistant port (5 mm) in the left flank region. The left lobe of the liver was retracted using a Nathanson liver retractor. The lesser sac was opened, and the greater omentum was divided preserving the gastroepiploic arcade. The stomach was mobilized completely after dividing the left gastric and gastroepiploic vessels (Figures 3a, 3b). The esophageal hiatus was opened, and communication established with the thoracic esophagus.

On the left side of neck, an oblique ‘J’-shaped incision was made anterior to the sternocleidomastoid muscle; esophagus was dissected and divided at the lower cervical level. An upper midline incision was given, and a gastric tube was prepared with multiple fires of linear cutting stapler with green cartridge. The specimen was retrieved through a small supraumbilical incision (Figure 3c), and the gastric conduit was pulled to the neck with help of a nasogastric tube fixed to the gastric tube. Hand-sewn side-to-side cervical esophagogastric anastomosis was done using polydioxanone (3-0) interrupted sutures. Neck wound was closed after approximating strap muscles and placing a Jackson-Pratt drain. Feeding jejunostomy (FJ) was done using the Witzel technique. After securing hemostasis, abdominal closure was done. The total blood loss was ~200 ml with an operative duration of six hours.

His postoperative course was uneventful with the FJ trial feed being given on postoperative day (POD) 1 and gradually increased which he tolerated well. Neck drain was removed on the second POD. An oral contrast study performed on the fourth POD showed no evidence of extravasation of contrast, following which he was started on liquids. Abdominal drain was removed on the fifth POD and he was discharged on the sixth POD on an oral semisolid diet. At the end of three months, he was eating comfortably and had no cough, gastroesophageal reflux or hoarseness of voice.

## Discussion

Corrosive-induced stricture of the esophagus (CISE) has been managed primarily by endoscopic dilatation, failing which surgery is considered as the treatment of choice. Esophagectomy in the elective setting for CISE is still controversial. The proponents of esophageal bypass in CISE reasoned that dissection of dense periesophageal adhesions can result in a high incidence of iatrogenic complications [1]. Further, the cumulative risk of malignancy within the retained esophagus is much less to merit risking morbidity linked to mediastinal dissection [1].

The proponents of esophagectomy argue that the risk of esophageal cancer in these patients has been reported to be 2%-3%, which is important given that most patients with CISE are young and at risk over a period of time [1,3,4]. In addition, the development of cicatricial carcinoma in the native esophagus is a silent process, as these patients do not develop the classical symptom of dysphagia unless it obstructs the anastomotic site and endoscopic surveillance of native esophagus is also not feasible [4]. Kim et al. reported esophageal cancer in seven out of 54 patients (13%) in the native esophagus with the interval between the injury and cancer being 29-46 years [1]. Hence, esophagectomy in CISE may be beneficial in young population where preserving the injured esophagus for long periods may predispose to malignancy. Furthermore, there always remains the risk of mucocele formation in the native esophagus [1,5]. It is more pronounced when there is a short-segment stricture in the middle or lower esophagus leaving a significant length of normal mucus secreting mucosa. Also, esophagectomy done in cases where gastric conduit is used has better outcome in terms of operative duration, blood loss, conduit ischemia and inhospital deaths as compared to colonic transposition [4]. Stomach as a conduit requires a single anastomosis, thereby decreasing the extra anastomosis and the consequent leak rates. In the present case, stomach was also preferred as it is the conduit of choice in low strictures [6].

Open thoracotomy or transhiatal esophagectomy (THE) has been performed for resection of the scarred esophagus or the subsequent mucocele [5,7]. The transthoracic approach, although easier, is invasive and associated with significant pulmonary complications. THE, on the other hand, is a blind approach and increases the risk of trauma to adjacent vital structures such as the trachea, aorta and pericardium, due to the dense periesophageal adhesions. In order to circumvent these drawbacks, laparoscopic-assisted THE had been proposed, but the extent of dissection remains limited (lower third of esophagus) with this approach [8]. Video-assisted thoracoscopic surgery, now, has become the standard of care in malignancy of esophagus, and extrapolation of this technique for treating CISE has been reported in anecdotal reports with positive results [2,9,10].

When esophagectomy is planned, the thoracoscopic approach in CISE has the following benefits: (a) prone position with addition of DLT helps in collapse of the lung allowing more room for easy periesophageal dissection; (b) maintaining resection plane close to the esophagus during the thoracoscopic phase under magnified vision prevents injury to vital mediastinal structures and potential complications compared to THE, which is a relatively blind procedure and has risk of injury to adjacent vital structures; (c) pain-free recovery with decreased pulmonary complications and early discharge as compared to a thoracotomy; and (d) the use of orthotopic route for the conduit reduces anastomotic complications, as it is the shortest possible route with easy performance of subsequent endoscopies. Furthermore, it reduces the chances of kinking and redundancy of the conduit [4,6].

## Conclusions

In the present case, thoracoscopic esophagectomy with laparoscopic gastric mobilization was successfully done with minimal morbidity. Hence, esophagectomy with gastric pull up in corrosive strictures can be safely accomplished using minimally invasive techniques especially in young patients. The morbidity of the minimally invasive technique compared with other approaches needs to be ascertained in a prospective trial.
